# Current therapies and mortality in acromegaly

**Published:** 2015

**Authors:** S Găloiu, C Poiană

**Affiliations:** * “C.I. Parhon“ National Institute of Endocrinology; ** “Carol Davila“ University of Medicine and Pharmacy, Bucharest, Romania

**Keywords:** acromegaly, mortality, medical therapy, pituitary surgery, somatostatin analogs

## Abstract

Acromegaly is a rare disease most frequently due to a GH secreting pituitary adenoma. Without an appropriate therapy, life of patients with acromegaly can be shortened with ten years. Pituitary surgery is usually the first line therapy for GH secreting pituitary adenomas. A meta-analysis proved that mortality is much lower in operated patients, even uncured, than the entire group of patients and is similar with the general population in patients with GH<1 μg/ L. For the patients with hypersecreting postoperative remnant tumor, those with low chance of surgical cure or with life-threatening comorbidities, medical therapies are available: somatostatin receptor analogues (SRA), dopamine agonists (DA) and GH receptor antagonists. Studies with >30% utilization of SRAs reported a lower mortality ratio than studies with lower percentages of SRA administration. Although therapy with DA has long been used in patients with acromegaly, there are no studies reporting its effect on mortality, but its efficacy is limited by the low remission rate obtained. The use of conventional external radiotherapy, although with good remission rate in time, was linked with increased mortality, mostly due to cerebrovascular diseases.

Conclusion. Mortality in acromegaly can be reduced to expected levels from general population by using modern therapies either in monotherapy or by using multimodal approaches in experienced centers.

## Introduction

Acromegaly is a rare disease most frequently due to a growth hormone (GH) secreting pituitary adenoma. Although usually with an indolent, slow evolution, diagnosed 5 years after first signs of acromegaly [**1**], cardiovascular and metabolic comorbidities are accompanied with an increased mortality in untreated patients. Without an appropriate therapy, life of patients with acromegaly can be shortened with ten years [**2**]. Before current therapies became available, the standard mortality rate (SMR) for these patients was reported to be of 2-3 [**3**]. However, in some studies there are meta-analyses reporting mortality similar with the general population in patients with normal GH after successful therapy and even normal insulin-like growth hormone 1 (IGF1) [**4**]. Using sensitive assays, cut-off level for “safe” GH is still a matter of debate, but recent consensus suggested this could be of 1 ng/ mL [**5**].

**Pituitary surgery**

Current guidelines suggest pituitary surgery to be the first line therapy for GH secreting pituitary adenomas, especially when experienced neurosurgeons are available and tumor is small and well delimitated [**6**]. Surgery related deaths are very rare nowadays. In experienced centers, the cure rate of surgery in macroadenomas achieved up to 74% cured patients, depending on GH criteria of cure and the technique used [**7**]. One study reported a better surgical result with an endoscopic approach, versus microscopic neurosurgery in patients with macroadenomas and suprasellar extension [**8**]. However, the success rate in other centers is much lower [**9**].

The impact of pituitary surgery on mortality depends on the ability of the neurosurgeon to remove the tumor, and this procedure is best performed in dedicated neurosurgical centers. Even in experienced centers, the long term follow-up, of up to 10 years, found a lower cure rate of patients initially considered successfully operated [**10**]. In a meta-analysis, Dekkers reported an overall standard mortality ratio (SMR) of 1.72 in all studied patients (n=16 studies), 1.32 in operated patients by transsphenoidal approach and 1.09 in patients with GH<1 μg/ L, similar with the reference population. The best predictor markers of mortality in operated patients were found to be GH at last follow up, GH three years postoperatively and IGF1 levels at last follow up [**11**]. An elevated GH level in the first three years post-surgery could be a better predictor factor for mortality than IGF1, probably due to the lack of standardization of IGF1 tests, lack of availability of IGF1 levels in all patients, susceptibility of interferences from binding proteins. In patients with discordant values of GH and IGF1 levels, the authors found that the benefits of reducing GH to <2 μg/ L outweigh the benefits of normalizing IGF1 levels. Behind these data, there are studies reporting better surgery results in patients with acromegaly pre-treated with somatostatin receptor analogues (SRA) [**12**], while others did not find such differences [**13**].

**Medical therapy**

For the patients with hypersecreting postoperative remnant tumor, those with low chance of surgical cure (i.e. cavernous sinus invasion) or with comorbidities that lead to contraindication of operation, medical therapies are available, grouped into three classes: somatostatin receptor analogues, dopamine agonists (DA) and GH receptor antagonists.

**Somatostatin receptor analogues**

SRA are usually the first line medical therapy indicated in acromegaly. Biochemical control rate reaches 60% in selected patients [**14**], but is usually 30% in unselected patients using strict criteria of GH and IGF1 normalization [**15**,**16**]. Long-term treatment (i.e. 10-15 years) with SRA, improves the biochemical response rate up to 80% of the patients [**12**]. Also, the dose optimization increases the rate of responders to medical treatment in patients with inadequate biochemical control [**17**]. Clinically significant tumor shrinkage (>20%) is obtained in 75% of the patients [**18**,**19**] and also rebound regrowth of pituitary tumor has been found after discontinuing treatment even in patients with inadequate biochemical control [**20**]. Currently available SRA: Octreotide LAR and Lanreotide Autogel have been reported to be similar in efficacy in both biochemical and tumor control [**21**,**22**] and slightly better than Lanreotide SR.

SRA can be administered as primary therapy in patients with stable macroadenomas without chiasm compression, in poor surgical candidates or who refuse surgery. There were studies reporting a better acromegaly outcome in terms of better GH and/ or IGF1 control and tumor volume reduction in patients treated with SRA before surgery. The first randomized, controlled study of preoperative Octreotide treatment of acromegaly (the POTA study) in 2008 concluded that patients with macroadenoma treated 6 months with Octreotide s.c. had a better cure rate at the 3 months post-operatory. However, a non-significant impact of this approach was observed in the same cohort followed at 1 and 5 years after surgery [**23**]. A meta-analysis published in 2013 enrolling almost 1000 patients from 10 studies revealed a significant effect of SRA pretreatment, on outcome only when analyzed the 3 RCT as a group (OR 3.62, 95% CI, 1.88-6.96) [**24**]. In contrast with this, in a randomized controlled trial, Colao did not find any difference in patients submitted to surgery or treated with SRA [**25**]. Moreover, a recent meta-analysis enrolling 2629 patients from 39 studies found a higher remission rate at the longest follow-up (i.e.>24 months) in patients submitted to surgery compared to medical treatment (67% vs. 45%) [**26**]. These data supported the current Endocrine Society acromegaly guideline recommending primary medical therapy in patients with severe pharyngeal thickness and sleep apnea, high-output heart failure and suggesting against the routine use of preoperative medical therapy to improve biochemical control after surgery [**13**]. On the contrary, surgical debulking has been found to improve the rate of response to medical treatment [**27**].

A promising new therapy for patients with acromegaly resistant to commercial available SRA is pasireotide, a multireceptor-targeted somatostatin analog with high affinity for 4 of the 5 somatostatin receptor subtypes (sst), including sst2 and sst5, which are the most prevalent sst on GH-secreting pituitary adenomas. One randomized, controlled trial compared the efficacy of pasireotide LAR and Octreotide LAR in naïve to medical therapy. Pasireotide LAR provided a strong suppression of IGF-1, and patients were 63% more likely to achieve biochemical control with pasireotide LAR than with Octreotide LAR, both in post-surgery and in de novo patients [**28**]. The profile of safety in the study was similar with other SRA, but hyperglycemia was 35% more frequent in pasireotide treated arm than in Octreotide treated arm. 

There are no conclusive data in literature regarding the effect of SRA treatment on mortality in patients with acromegaly. One study compared the effect of surgery and medical therapy on mortality in Italian patients with acromegaly. Surgery resulted in a better survival rate than medically treated patients, but there was a selection bias in this study, patients treated with SRA being more likely to have comorbidities making them more unsuitable for surgery [**29**]. A meta-analysis by Holdaway et al. demonstrated that studies with >30% utilization of SRAs or >70% of remission rates reported lower SMR (1.7, CI (1.5-2)) than studies with lower percentages of SRA administration and remission rates (SMR=2, CI 1.6-2.3) [**4**].

**Dopamine agonist therapy**

The mostly used DA is cabergoline, which leads to the normalization of GH and IGF1 in 27% of the patients [**30**]. It may exert antiproliferative and pro-apoptotic effects on GH secreting tumors, ACTH secreting tumors, besides known effects on prolactinomas and it has the advantage of oral administration and low cost compared to SRA or GH receptor antagonists. A large multicenter study in Belgium reported IGF1 reduction in 39% of the patients and GH<2 μg/ L in 46% of the patients treated with cabergoline monotherapy. Better results were obtained in patients with mixed GH and PRL secreting pituitary tumors and in those with marginally elevated GH/ IGF1 levels [**31**]. In another study on patients from the Bulgarian Acromegaly database, control rates on cabergoline therapy were 18.2% and on bromocriptine 16.3%, in patients without prior radiotherapy. Predictors of response to dopamine agonist therapy were active acromegaly duration, radiotherapy and medication dose [**32**]. There are safety issues regarding the long-term treatment with cabergoline: patients with Parkinson disease treated with high dose of cabergoline were reported to have an increased risk for severe valvular regurgitation [**33**]. There are no data regarding clinically significant valvular regurgitations in patients with acromegaly treated with cabergoline, acromegaly itself raising the risk of valvular disease. Also, one study showed that impulse control disorders are 9.9 times more common in male patients with prolactinomas taking DA than in the control group patients [**34**].

Although this therapy has been long used in patients with acromegaly, there are no studies reporting the effect of monotherapy with DA on mortality. One study compared patients from Bulgaria, with low cure rate of surgery, high usage of radiotherapy and dopamine agonists and usage of SSA only after 2008 with patients from Campania, Italy and life expectancy was significantly shorter in patients from Bulgaria [**35**].

**GH receptor antagonist therapy**

Pegvisomant is a genetically modified analog of human GH and acts as a competitive GH receptor antagonist. Biochemical control is obtained in 75-92% of the patients from randomized control trials [**36**]. Its long-term safety (mean 3.7 years, range - 0 to 12.5 years) has been studied in 1288 patients from ACROSTUDY cohort: a central MRI reading found a 3.2% increase/ decrease in tumor size from 936 patients with at least two available MRI. 2.5% of the patients had liver enzyme increases greater than 3 times the upper limit of normal. Mortality ratio was 1.2% during the study; the most frequent cause of death was heart failure and cancer related.

**Combination therapy**

In patients with partial resistance to SSA, cabergoline adding could provide a better control rate, independent of prolactin levels, especially in those with mild elevated GH levels [**30**]. Also, a weekly dose of 60-80 mg pegvisomant in association with a monthly dose of SSA improve the response rate of both therapies to 95% [**37**]. Pegvisomant can also be used in combination with cabergoline therapy, combination therapy proving a better acromegaly control than monotherapy with either medication [**38**].

Estrogens, selective estrogen receptor modulators (SERMs), alone or in combination with SSA, have been observed to decrease IGF-1 in women and to improve symptoms of acromegaly [**39**]. Clomiphene citrate, a SERM usually used for ovulation induction, was successfully used in men with acromegaly not controlled after surgery, radiotherapy and conventional medical treatment and with testosterone level within or below the third inferior tertile of normality, with an additionally increase of testosterone level [**40**]. 

Radiotherapy is now considered an adjuvant therapy in uncontrolled patients with acromegaly after pituitary surgery and medical therapy [**41**]. Remission rate of conventional radiotherapy defined as serum basal GH levels <2.5 μg/ L reported is up to 77% after 20 years [**42**]. Its efficacy is tempered by long time needed until radiotherapy became effective and by concerns about long-term toxicity: second tumor induction, mortality due to cerebrovascular disease in patients already exposed to a high risk of vascular diseases as proved by carotid artery modifications. A study on 462 patients with pituitary adenomas, including 139 patients with acromegaly, found no differences between intracranial tumors incidence and no death events in patients with postoperative radiotherapy as compared to patients submitted to surgery alone [**43**].

The use of external radiotherapy was linked with increased mortality, with a SMR of 1.58 in the West Midlands Acromegaly Study. Also, in the Finnish Nationwide Survey of Mortality in Acromegaly, treatment with radiotherapy was one of the determinants of mortality, but with borderline statistically significance [**44**]. The new techniques of radiotherapy: intensity modulated RT (IMRT), fractionated stereotactic radiosurgery (FSR), stereotactic radiosurgery (SR), and proton beam techniques have promising results. Rates of hypopituitarism ranged from 28 to 80% in patients with pituitary adenomas, depending on the time from the pituitary irradiation. Hypopituitarism is another factor contributing to the increased mortality rate after radiotherapy, its incidence being more reduced with new focused modalities of radiotherapy [**45**]. 

## Conclusion

Mortality in acromegaly can be reduced to expected levels from general population by using modern therapies either in monotherapy or by using multimodal approaches in experienced centers.

**Acknowledgement**

”This work received financial support through the project entitled “CERO – Career profile: Romanian Researcher”, grant number POSDRU/159/1.5/S/135760, cofinanced by the European Social Fund for Sectoral Operational Programme Human Resources Development 2007-2013”.

**Conflict of interest**

The authors declare there were no conflicts of interest.
